# Contrasting response of polyamine metabolic enzymes underlying fiber-type specific volume regulation in the skeletal muscle with endurance exercise

**DOI:** 10.1016/j.jphyss.2026.100083

**Published:** 2026-06-17

**Authors:** Maki Yamaguchi, Makiko Ohkido, Naoya Nakahara, Kazuhiro Hirano, Shigeru Morimoto, Michiaki Ikeda, Toshiko Yamazawa, Hideki Yamauchi

**Affiliations:** a The Jikei University School of Medicine, Department of Molecular Physiology, Japan; b The Jikei University School of Medicine, Department of Molecular Biology, Japan; c The Jikei University School of Medicine, Core Research Facilities, Japan

**Keywords:** Hypertrophy, Polyamine, Putrescine, Endurance training, Aerobic metabolism

## Abstract

To explore the role of polyamine metabolism in muscle-type-specific hypertrophy, we analyzed changes in the expression profiles of polyamine metabolic enzymes and key enzymes for muscle metabolism induced by voluntary wheel running exercise in different muscle types. Effect of the polyamine precursor, putrescine, which was reported to have potential to regulate muscle volume, was also tested. Polyamine synthetic enzymes were upregulated in hypertrophic soleus muscle whereas polyamine catabolic enzymes were upregulated in non-hypertrophic plantar muscle by exercise in correlation with increased mitochondria-related protein expression. The increased catabolic enzymes of polyamines in the plantar muscle were hypothesized to be possibly involved in restriction of hypertrophy in fast-type skeletal muscles correlating with increased aerobic metabolism. Putrescine administration minimally affected polyamine metabolism and muscle volume indicating that it did not effectively regulate muscle hypertrophy. Polyamine oxidase localized in the perinuclear and inter-myofibrillar region suggesting a correlation between aerobic metabolism and polyamine catabolism.

## Highlights

• Polyamine catabolism accelerated in non-hypertrophic fast type muscle by exercise.
• Aerobic metabolism remarkably accelerated in fast type muscle by exercise.
• Polyamine oxidase distributed in a similar pattern to mitochondria.
• Involvement of polyamine catabolism and aerobic metabolism was implied.

## Graphical Abstract

**Figure:**
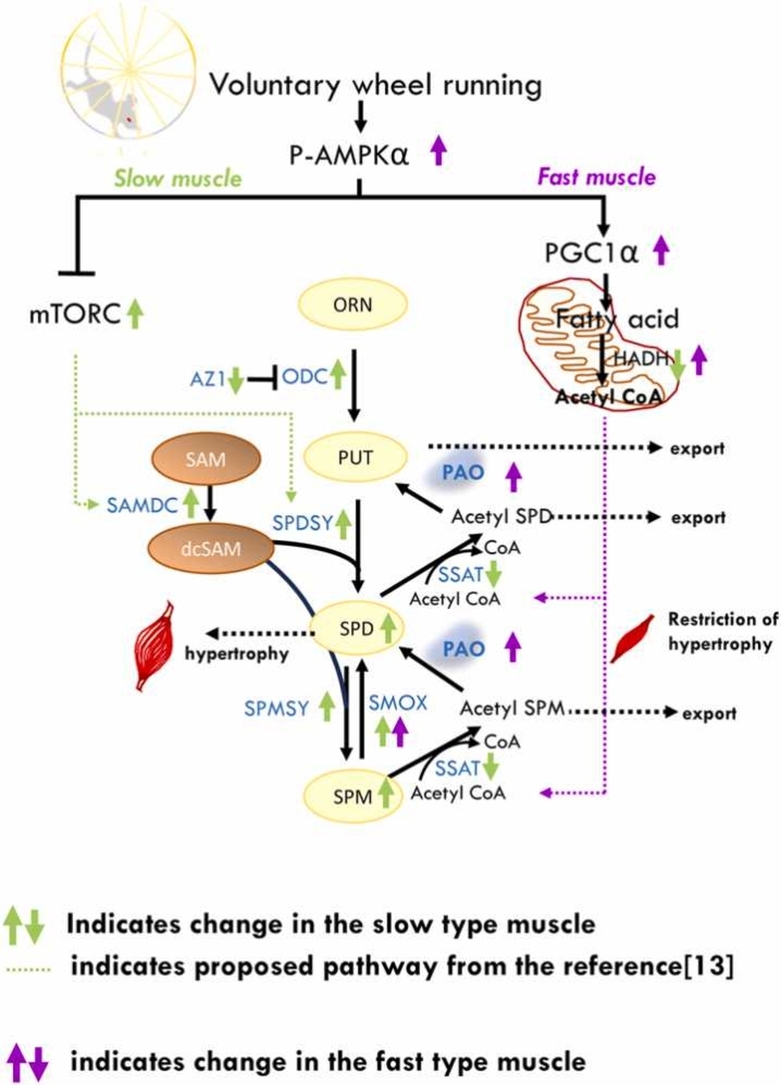
Summary of the main results in this study and possible relationship between polyamine metabolism and conventional pathways associated with hypertrophy. Solid arrows show change of the direction of protein expression in the soleus (light green) and plantar (magenta) muscle. Broken lines show possible pathways. The pathway from mTORC to SAMDC and SPDSY (shown in light green) was drawn according to the result from Tabbaa et al. (Reference 13).

## Introduction

Endurance exercise differently stimulates hypertrophy in muscles depending on the muscle type. Signaling pathways centered on AMPK are widely accepted as regulators of the different responses between muscle types. That is, in the fast-type muscle, endurance exercise stimulates AMPK phosphorylation efficiently, leading to significant activation of oxidative metabolic enzymes via the PGC1α signal pathway [1], [2]. Simultaneously, the mTORC pathway is inhibited by AMPK [3], [4] thereby suppressing downstream muscle hypertrophic responses. By contrast, AMPK phosphorylation is less likely to occur in the slow-type muscle, allowing mTORC-mediated hypertrophic response to proceed [5].

Polyamines are small cationic molecules that are prevalent in all living cells and are essential for versatile cell processes, including cell proliferation, protein synthesis, autophagy, oxidative metabolism, and antioxidant effects [6], [7], [8]. Polyamine metabolism is finely tuned under coordinated regulation by synthetic enzymes; ornithine decarboxylase (ODC), which is a key enzyme in de novo polyamine synthesis from ornithine; ODC antizyme (AZ), which inhibits the function of ODC; *S*-adenosylmethionine decarboxylase (SAMDC), which catalyzes the conversion of S-adenosyl methionine (SAM) to decarboxylated S-adenosyl methionine (dcSAM); spermidine synthase (SPDSY), which catalyzes the synthesis of spermidine from putrescine and dcSAM; and spermine synthase (SPMSY), which catalyzes the synthesis of spermine from spermidine and dcSAM, and catabolic enzymes; spermidine/spermine N1-acetyltransferase (SSAT), which catalyzes the acetylation of spermidine and spermine; polyamine oxidase (PAO), which catalyzes the conversion of acetylated spermidine/spermine to putrescine/spermidine; and spermine oxidase (SMOX), which catalyzes the direct oxidation of spermine to spermidine [9], [10], [11].

Several recent studies have demonstrated the regulation of the muscle volume and quality is involved with metabolism of polyamine [10], [12], [13], [14], [15], [16] and hypusinated eIF5A [17], [18], that is synthesized by a post-translational modification in which spermidine acts as an amino-butyl group donor. In this context, extensive research has been conducted on polyamine synthetic enzymes from the perspective of volume regulation in striated muscle and SAMDC and SPDSY have been clarified to be activated in mTORC1 dependent manner by the experiment using mTORC1 inhibitor [13]. However, the role of the catabolic enzymes of polyamine in the volume regulation of striated muscles remains unclear.

This study aimed to elucidate how polyamine metabolism responds to endurance exercise by comparing the expression profiles of polyamine metabolism and relevant key proteins in different types of skeletal muscles. We selected plantar muscles, largely composed of fast-glycolytic (FG) and fast-oxidative glycolytic (FOG) fibers, which preferentially induce metabolic adaptation rather than hypertrophy of muscle fibers [19], [20], [21], and soleus muscles, composed of FOG and slow-oxidative (SO) fibers, in which the hypertrophic response is remarkable, in response to endurance exercise.

We also evaluated the response of the cardiac muscle, which exhibits eccentric hypertrophy by endurance exercise, in which elongation of the muscle fiber is dominant over radial thickening of the fiber, resulting in ventricular enlargement [22], to compare the contribution of polyamine metabolism to different types of hypertrophic responses.

In addition, the effect of the polyamine precursor, putrescine, which has potential to stimulate hypertrophy of myotubes in vitro [16], was investigated to evaluate whether exogenous putrescine acts as a muscle volume regulator in vivo.

## Methods

### Experimental procedure and muscle tissue preparation for the main experiment

**Fig. 1:**
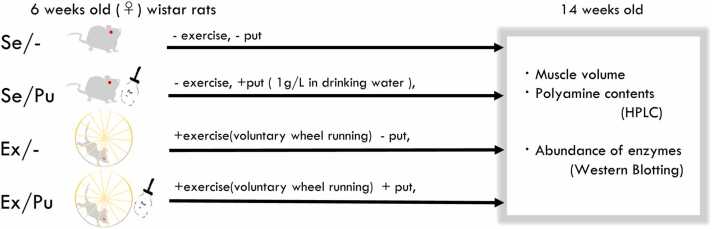
Graphical presentation of the experimental protocol. Detail of the animals and experimental protocol are provided in the text.

The animal experiments were reviewed and approved by the Institutional Animal Care and Use Committee of Jikei University (26−026) and conformed to the Guidelines for the Proper Conduct of Animal Experiments of the Science Council of Japan (2006). Six-week-old female Wistar rats were subjected to the exercise and putrescine administration (1 g/L in drinking water) experiments (Fig. 1). They were divided into four groups as follows: normal housing conditions with no application of putrescine or exercise (Se/−, n = 10), putrescine administration without exercise (Se/Pu, n = 9), exercise without putrescine administration (Ex/−, n = 9), and exercise with putrescine administration (Ex/Pu, n = 9). During the 8-week experimental period, the animals were provided water and food ad libitum. Exercise was provided by the wheel set in the cage with a load of 30% body weight [1], and animals were allowed to access the wheel freely. Body weight, food intake, water intake, and running distance were measured every 2–4 days. Within 2 weeks of the end of the experimental period, computed tomography images of each rat were obtained to evaluate the fat rate in the lumbar region from the second to forth lumbar vertebrate using a Latheta LCT−200 (Hitachi Aloka Medical. Ltd., Tokyo, Japan). The data on body weight, fat rate, food intake, water intake, putrescine intake, and running distance are summarized in Supplementary Table 1.

After the experimental period, the animals were euthanized by exsanguination from the groin under deep isoflurane anesthesia. After immediate excision of muscle tissues, the right side of the plantar muscle, soleus muscle, and the apex part of the cardiac muscle were stored at −80 °C to analyze polyamine contents using high-performance liquid chromatography (HPLC). The left side of the plantar and soleus muscles, and the remaining part of the cardiac ventricles were quickly frozen in liquid nitrogen and stored at −80 °C until western blotting analysis.

### Western blotting

Western blot analysis was performed. Each muscle specimen was minced and homogenized in a cold lysis buffer. The homogenate was gently stirred for 60 min at 4 °C and then centrifuged at 16,000 *g* for 20 min at 4 °C. The supernatant was collected, and the protein content was determined using the Lowry method with bovine serum albumin as a standard. The samples were solubilized in Laemmli sample buffer and boiled at 95° C for 5 min. Using a 7.5%–15% polyacrylamide gel, 20 μg of protein from each sample was separated by electrophoresis and subsequently transferred to a polyvinylidene difluoride membrane. After the transfer, the membranes were washed with Tris-buffered saline containing 0.1% Tween 20 (TBS-T) and blocked with 1% skim milk or 1% bovine serum albumin in TBS-T for 1 h at room temperature. After blocking, the membranes were washed and incubated overnight at 4 °C with primary antibodies (Supplementary Table 2). The membrane was washed with TBS-T and incubated with the appropriate secondary antibody (Cell Signaling Technology) for 1 h at room temperature. A chemiluminescent reagent (ImmunoStar Zeta or LD; Wako Pure Chemical, Osaka, Japan) was used to detect the protein bands. The bands were scanned using a chemiluminescence detector (LAS−3000 mini, Fujifilm, Tokyo, Japan), and their intensities were quantified using Multi Gauge (Fujifilm, Tokyo, Japan). The expression level of each protein was standardized to the density of the standard sample, which was prepared by mixing four samples for each gel. For the graph presentation of the expression level of each protein, the average value of each group was normalized to that of the control (Se/−) group.

### Measurement of putrescine and polyamine contents

Each frozen muscle specimen was cut into small pieces and homogenized in nine or four volumes of sterile distilled water using a high-speed universal homogenizer (Hiscotron; Microtech Nichion Corporation, Funabashi, Japan). An aliquot of homogenate for high-pressure liquid chromatography (HPLC) analysis was mixed with 40% perchloric acid, one-ninth the amount of aliquot, vortexed for 1 min, sonicated thrice for 10 s, kept on ice for 5 min, and centrifuged at 15,000 rpm at 4 °C for 15 min. The supernatant was filtered through a Millex-LH 0.45 μm (Merck Millipore) and subjected to HPLC analysis using a Shimadzu LC Solution System (LC−20AT, SIL−20AC, CTO−20A, and RF−10AXL). Putrescine and polyamines were detected using ortho-phthalaldehyde (Nacalai Tesque, Kyoto, Japan) after separation using a cation exchange column, Shim-pack ISC−05/S0504 (Shimadzu, Kyoto, Japan), as previously reported [23]. Putrescine and polyamine concentrations were determined from each peak area and normalized by dividing with the protein concentration measured using the Pierce bicinchoninic acid protein assay kit (Thermo Fisher Scientific Inc., Waltham, MA, USA).

### Confocal laser microscopic observation of muscle fiber

For structural analysis, muscle bundles were harvested from the soleus and plantar muscles of Wistar rats reared under the same conditions as the biochemical experiments for the same duration and fixed in phosphate-buffered saline containing 4% paraformaldehyde for 1 h to serve as samples. Single muscle fibers obtained from the muscles were used to analyze protein localization. Nonspecific binding of the antibody to specimens was blocked in a buffer containing 10% goat serum and 1% Triton X−100 for 3 h. Then specimens were treated with the primary antibodies with appropriate dilution in the 5% goat serum and 0.3% Triton X−100 for 18 h at 4 °C. Subsequently, they were treated with the fluorescent labeled goat anti-rabbit IgG, goat anti-mouse IgG_1_, and Propidium iodide diluted in 5% goat serum and 0.1% Triton X−100 for 18 h at 4 °C. Details of the chemicals used are summarized in Supplementary Table 2.

Confocal laser scanning microscopy images were acquired using a ZEISS LSM 980 laser scanning confocal microscope equipped with a Plan-Apochromat 20 × /0.8 or 63 × /1.40 objective and image acquisition software Zen 3.8 (Carl Zeiss, Jena, Germany) at the core facility center of the Jikei University School of Medicine. Fluorescence was excited with 405, 555, and 639 nm lasers and detected using GaAsp-PMT detectors with appropriate emission filters.

### Statistical analysis

Statistical analysis for comparison of the means of exercise and putrescine administration experiments was performed using a two-way analysis of variance (ANOVA) with factors of exercise and putrescine administration using Stat View ver 5.0. When an interaction was detected, one-way ANOVA and post-hoc tests were performed using the Tukey–Kramer test. Spearman’s rank correlation analysis between parameters was performed using R.4.5.0.

## Results

### Change in muscle volume and polyamine contents in skeletal muscles

**Fig. 2:**
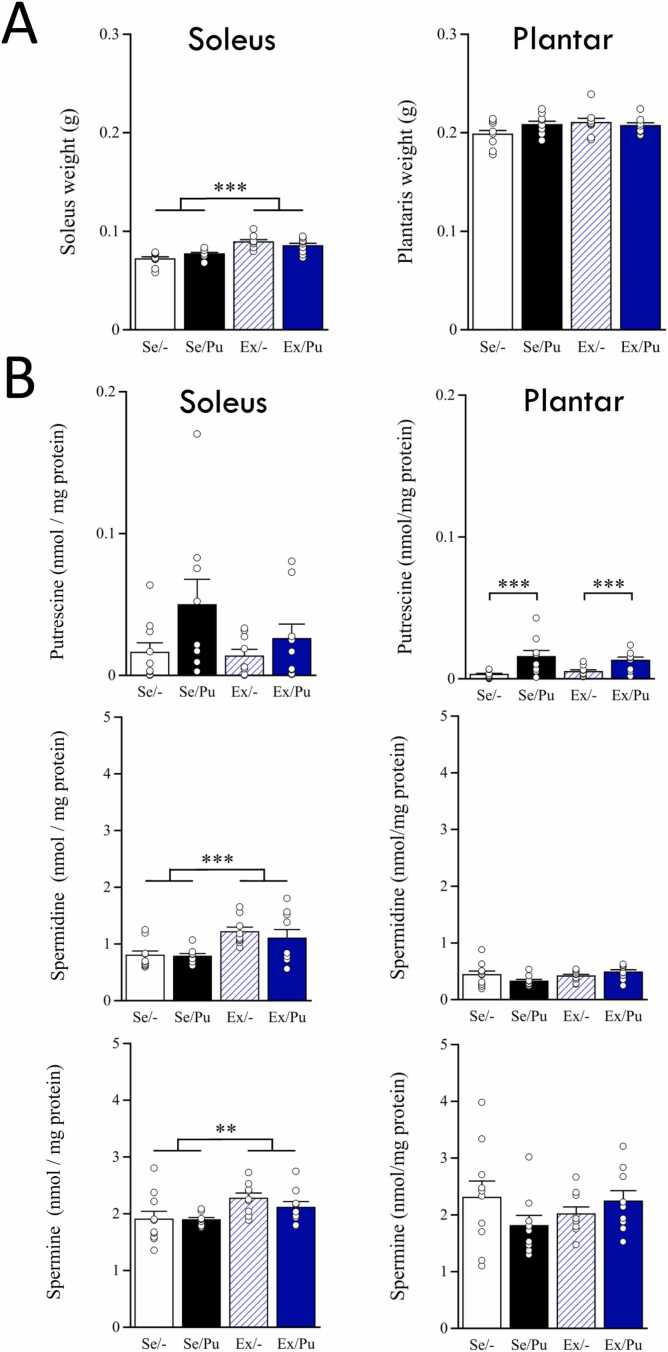
Change of muscle volume and polyamine contents. A. Muscle volume of plantar and soleus muscles after the experimental period for groups of sedentary without putrescine application (Se/−), sedentary with putrescine application (Se/Pu), exercised without putrescine application (Ex/−), exercised with putrescine application (Ex/Pu). Data are presented as means ± standard errors of the mean (S.E.M.). *** indicates p < 0.001 by two-way analysis of variance (ANOVA) for the factor of exercise application. B. Contents of putrescine, spermidine, and spermine in the plantar and soleus muscles after the 8-week experiment for sedentary without putrescine application (Se/−), sedentary with putrescine application (Se/Pu), exercised without putrescine application (Ex/−), and exercised with putrescine application (Ex/Pu) groups. Data are indicated as means ± standard errors of the mean (S.E.M.). ** and *** indicate p < 0.01 and p < 0.001 by two-way analysis of variance (ANOVA) for the factor of putrescine administration (for the putrescine content in the plantar muscle) or exercise application (for others), respectively.

Voluntary wheel running application induced approximately 20% change in the weight of the soleus muscle, consisting of slow oxidative (SO) type I and fast oxidative glycolytic (FOG) type IIa fibers, with strong correlation with the total running distance, irrespective of the putrescine intake (Fig. 2A and S1). Meanwhile, in the plantar muscle consisting of a large population (approximately 45%) of fast glycolytic (FG) type IIb fibers as well as FOG type IIx/IIa (ca.50%) and I (ca.5%) [24], muscle weight did not change either by exercise or putrescine administration. In the cardiac muscle, which consists of type I fibers exclusively, approximately 10% in the weight changed, irrespective of the putrescine intake (Fig. S2). These results are consistent with those of previous studies that reported that endurance training differentially induces hypertrophy depending on the muscle fiber type [25].

Polyamine and putrescine contents exhibited different responses in the muscles　(Fig. 2B). Putrescine administration increased the putrescine content in the plantar muscle, irrespective of exercise. In the soleus muscle, putrescine administration accumulated putrescine under sedentary conditions; however, it was weakened when exercise was applied (Fig. 2B).

The average contents of spermidine and spermine increased in the soleus muscle, positively correlating with the total running distance, but they did not change in the plantar muscle (Fig. 2B and S1).

### Change of polyamine metabolic enzymes in skeletal muscles

**Fig. 3:**
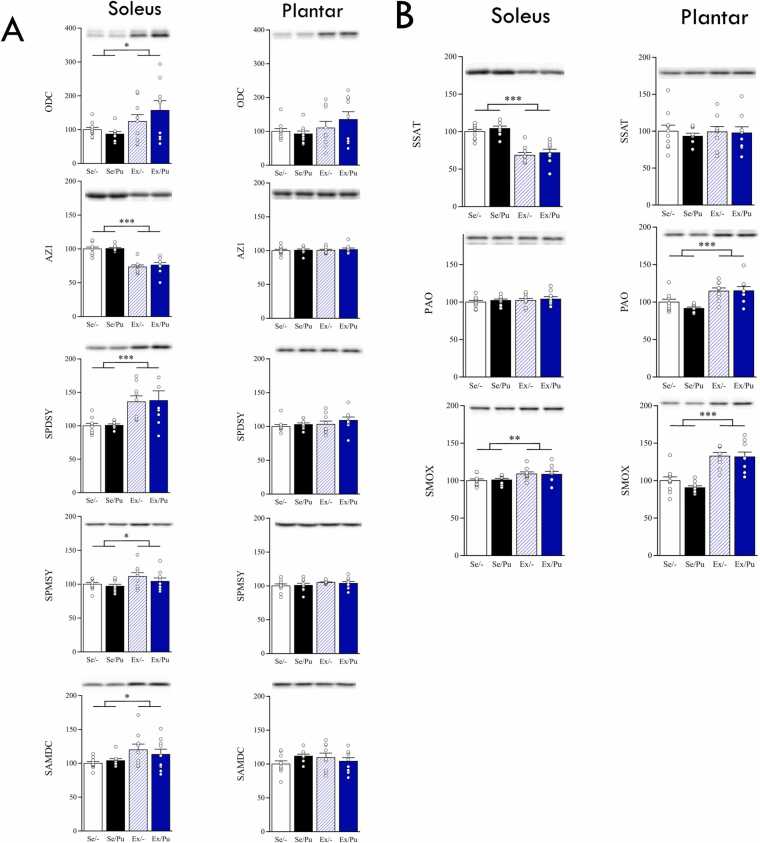
Change of polyamine metabolic enzymes. A. Averaged abundance of ODC, AZ1, SPDSY, SPMSY, and SAMDC in the plantar and soleus muscles after the 8-week experiment for groups of sedentary without putrescine application (Se/−), sedentary with putrescine application (Se/Pu), exercised without putrescine application (Ex/−), exercised with putrescine application (Ex/Pu). Data are presented as means ± standard errors of the mean (S.E.M.). * and *** indicate p < 0.05 and p < 0.001 by two-way analysis of variance (ANOVA) for the factor of exercise application, respectively. B. Average abundance of SSAT, PAO, and SMOX in the plantar and soleus muscles after the 8-week experiment in the sedentary groups without putrescine application (Se/−), sedentary with putrescine application (Se/Pu), exercised without putrescine application (Ex/−), and exercised with putrescine application (Ex/Pu). Data are presented as means ± standard errors of the mean (S.E.M.). ** and *** indicate p < 0.01 and p < 0.001 by two-way analysis of variance (ANOVA) for the factor of exercise application, respectively.

In the soleus muscle, the average abundance of the polyamine synthetic enzymes ODC, SAMDC, SPDSY, and SPMSY significantly increased positively correlating with the total running distance. Furthermore, the abundance of AZ1 was consistently inhibited by the exercise. Thus, polyamine metabolism was directed toward polyamine synthesis in the soleus muscle, accompanied with a hypertrophic response by the exercise, as anticipated in earlier studies [12]. By contrast, the average abundance of polyamine synthesis enzymes did not change in the plantar muscle (Fig. 3A).

The average abundance of polyamine catabolic enzymes also differently changed in each muscle types (Fig. 3B). In the soleus, SSAT decreased with a strong negative correlation with the total running distance (Fig. S1). SMOX levels increased slightly, whereas PAO levels did not change. By contrast, in the plantar muscle, PAO and SMOX remarkably increased and were strongly correlated with the total running distance. Thus, polyamine metabolism was clearly directed toward synthesis by coordinated changes in metabolic enzymes in the hypertrophic soleus muscle, whereas it was directed toward catabolism in the plantar muscle by the exercise.

### Change of key signaling molecules in skeletal muscles

**Fig. 4:**
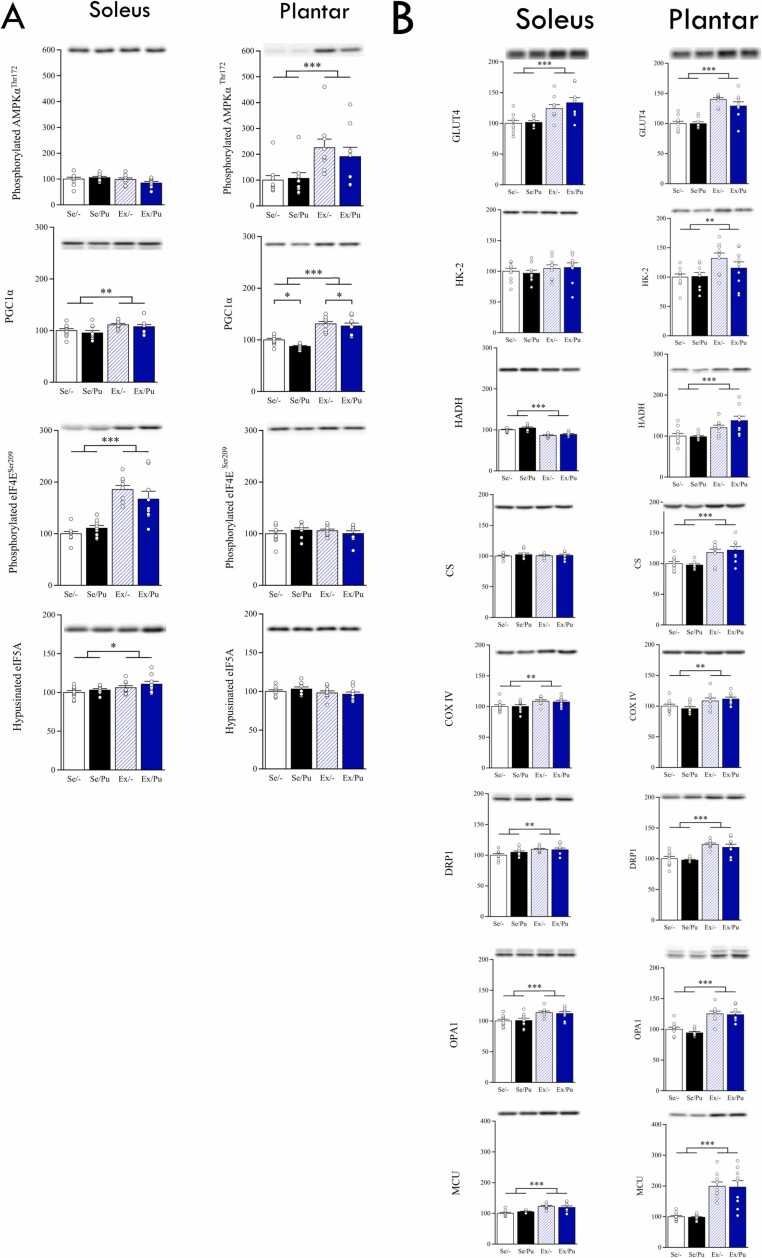
Change of the enzymes relating to AMPK–PGC1 axis and oxidative metabolism. A. Average abundance of phosphorylated AMPKα^Thr172^, PGC1α, phosphorylated eIF4E^Ser209^, and hypusinated eIF5A in the plantar and soleus muscle after the 8-week experiment for groups of sedentary without putrescine application (Se/−), sedentary with putrescine application (Se/Pu), exercised without putrescine application (Ex/−), exercised with putrescine application (Ex/Pu). Data are presented as means ± standard errors of the mean (S.E.M.). *, **, and *** indicate p < 0.05, p < 0.01, and p < 0.001 by two-way analysis of variance (ANOVA) for the factor of putrescine administration (for the PGC1α in the plantar muscle) or exercise application (for the PGC1α in the plantar muscle and others), respectively. B. Expression levels of enzymes related to mitochondrial respiration in the plantar and soleus muscles after the 8-week experiment for sedentary without putrescine application (Se/−), sedentary with putrescine application (Se/Pu), exercised without putrescine application (Ex/−), and exercised with putrescine application (Ex/Pu) groups. Data are presented as means ± standard errors of the mean (S.E.M.). ** and *** indicate p < 0.01 and p < 0.001 by two-way analysis of variance (ANOVA) for the factor of exercise application, respectively.

Signaling molecules which play crucial role for muscle metabolism differently changed in response to exercise (Fig. 4A). Phosphorylated species of AMPKα, which inhibits mTORC activity, increased with exercise in the plantar muscle. This is consistent with the report that increased metabolic stress due to deficiency of oxidative metabolic capacity in FG fibers stimulates AMPKα phosphorylation [2]. While in the soleus muscle, phosphorylated species of eIF4E, an index of the activation level of mTORC and considered to be inhibited by phosphorylated AMPKα, increased by the exercise, consistent with the lack of increase in phosphorylated AMPKα. The hypusinated form of eIF5A increased in the soleus muscle but not in the plantar muscle, in parallel with the change in spermidine levels by the exercise.

Abundance of PGC1α, key inducer of oxidative metabolic enzymes, which is induced by phosphorylated AMPKα, was remarkably upregulated by the exercise in the plantar muscle, consistent with previous findings [26], [27], [28]. Unexpectedly, abundance of PGC1α was suppressed by putrescine administration in the plantar muscle. Also, in plantar muscle, GLUT4 and HK−2, which promote glucose uptake and utilization, HADH, which promotes β-oxidation of fatty acid, were increased by the exercise (Fig. 4B), suggesting that lipids and sugar degradation to produce substrate for energy production was facilitated. In addition, mitochondrial enzymes catalyzing aerobic energy production, CS and COXIV, and the regulators of mitochondrial function and homeostasis, DRP1, OPA1, and MCU were also remarkably upregulated in the plantar muscle by the exercise. Abundance of PGC1α and mitochondria-related enzymes strongly correlated with PAO (Fig. S1) in the plantar muscle, suggesting a close relationship between polyamine catabolic enzymes and mitochondrial function. Meanwhile, in the soleus muscles, although GLUT4 increased, CS did not change and HADH was even downregulated suggesting suppressed β-oxidation of fatty acid (Figs. 4B and S1). Mitochondria-related enzymes, COX IV, DRP1, OPA1, and MCU increased their abundance, but the rate of increase was mild compared with plantar muscle.

### Localization of polyamine catabolic enzymes within skeletal muscles

**Fig. 5:**
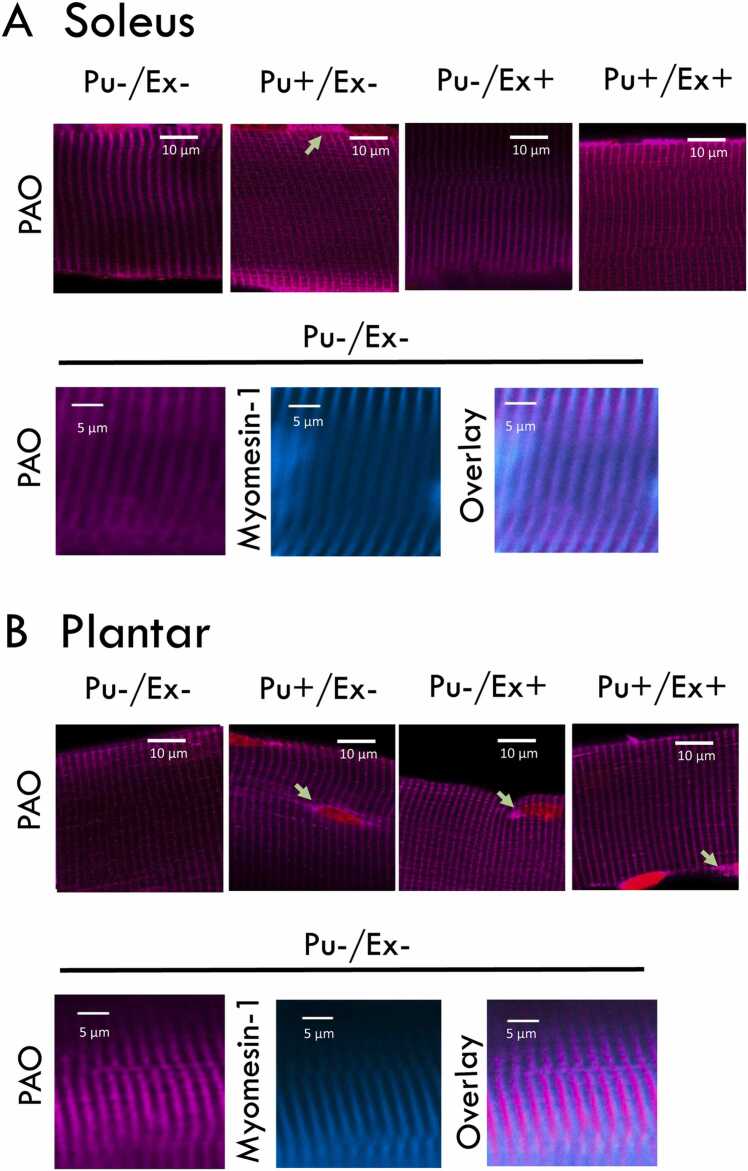
Confocal laser scanning microscopic images of a single muscle fiber. A: (Upper) Confocal laser scanning microscopy images obtained by immunostaining of single muscle fibers from the soleus muscle. The fibers were stained with rabbit IgG against PAO and visualized using fluorescently labeled anti-rabbit IgG and propidium iodide. Arrows indicate perinuclear accumulation of PAO. (Lower) Fluorescent signal from PAO (left), myomesin−1 (middle), and overlayed (right) images are shown. The details of the chemicals used are listed in Supplementary Table 2. B: (Upper) Confocal laser scanning microscopiy images obtained by immunostaining of single muscle fibers from the plantar muscle. The fibers were stained with rabbit IgG against PAO and visualized using fluorescently labeled anti-rabbit IgG and propidium iodide. Arrows indicate perinuclear accumulation of PAO. (Lower) Fluorescent signals from the PAO (left), myomesin−1 (middle), and overlaid (right) images are shown. The details of the chemicals used are listed in Supplementary Table 2.

To obtain structural insight into association between mitochondria function and polyamine catabolic enzymes, we evaluated the intracellular localization of PAO within the muscle fiber which was distinctively upregulated in the plantar muscle. Confocal laser microscopic analysis of skeletal muscle fibers revealed that the PAO was localized to the perinuclear region as well as the intermyofibrillar region with a striated distribution along the longitudinal axis in the plantar and soleus muscles in all experimental groups. Double staining with myomesin−1, which resides in the middle of the A-band, showed an alternating arrangement with PAO suggesting that the striated distribution of PAO would be at the Z-band (Fig. 5). Considering that mitochondria within muscle fibers are also localized in the perinuclear and intermyofibrillar regions, including the Z-band region [29], this result suggests the close relationship between polyamine catabolism and mitochondria.

### Change of polyamine metabolic enzymes and relevant proteins in the cardiac muscles

In the cardiac muscle, spermidine content unexpectedly decreased negatively correlating with the total running distance (Figs. S1 and S2). The average abundance of polyamine metabolic enzymes was hardly affected by exercise or putrescine administration, except for AZ1, which increased with putrescine intake, irrespective of exercise. Some of the enzymes related to energy metabolism (HK−2, HADH, DRP1, and OPA1) showed mild change of the abundance by the exercise. The hypusinated form of eIF5A increased in the cardiac muscle in a manner opposite to the change of spermidine content.

## Discussion

### Enhanced polyamine synthesis in the soleus muscle in response to the exercise

In the soleus muscle, which showed significant hypertrophy, polyamine content increased accompanied with the coordinated change of metabolic enzymes as though they respond to elevation of the “set point “of polyamine level. Regarding the upregulation of polyamine synthesis, Tabbaa et al. have reported that SAMDC and SPDSY upregulation is associated with muscle hypertrophy induced by mechanical overload with myotenectomy [13]. Since the upregulation of these enzymes is effectively inhibited by an mTORC1 inhibitor, the upregulation of polyamine synthetic enzymes also observed in this study would act as downstream factors of the mTORC pathway, at least partially. As for the SSAT, which was significantly decreased in this study in the hypertrophic soleus muscle, no change was observed in their experimental condition. The factors upstream of suppression of polyamine catabolic enzymes in the hypertrophic muscle will be elucidated through comprehensive experiments on the signaling pathways.

### Enhanced polyamine catabolism in the plantar muscle in response to the exercise

In contrast, in the plantar muscle, polyamine metabolism was directed toward catabolism to produce the acetylated form or low-amino group polyamines. Increased PAO expression in the plantar muscle induced by endurance exercise is remarkable, as the flux through PAO activity is usually regulated by the availability of substrates provided by SSAT rather than by changes in PAO activity [30]. The upregulation of PAO expression in the plantar muscle due to endurance exercise and the fact that acetylated polyamine and putrescine are relatively excretory from the cells [10], [11] suggest that the enhanced catabolism in plantar muscle may act to maintain the low polyamine concentration or hypusinated eIF5A. The enhanced catabolism of polyamines may contribute to restricted hypertrophic response in the fast-type muscle to endurance exercise, in addition to suppression of mTORC pathway by the increased phosphorylation of AMPK [3], [4].

One may wonder why polyamine levels, especially spermine, do not decrease with exercise despite the upregulation of polyamine catabolic enzymes which convert polyamines to low-amino group species in the plantar muscle. In relation to this point, it is notable that polyamine content within the cells is in a balance between supply and degradation from polyamine pool in the body [6]. Therefore, we consider that possible increase in polyamine influx may replenish polyamine in the plantar muscles thereby maintaining polyamine concentration.

### Postulated relationship between polyamine catabolism and aerobic metabolism

Overexpression of SSAT has been reported to reduce acetyl-CoA and promote β-oxidation of fatty acid [31], [32]. Thus, polyamine metabolism and aerobic metabolism could be crucially linked via acetyl-CoA. From this perspective, it may be postulated that the promotion of fatty acid β-oxidation, as indicated by the HADH upregulation, may elevate acetyl-CoA production in the plantar muscle. The increased production of acetyl Co-A could possibly enhance acetyl polyamine synthesis via SSAT, thereby upregulating the PAO expression in a substrate-induced manner.

A possible relationship between the protein expression of HADH and polyamine catabolic enzymes was also observed in the soleus muscles in those abundances of HADH and SSAT showed a strong positive correlation (Fig. S1). Although we cannot elucidate the causal relationship between the fatty-acid oxidation and polyamine catabolism in this study, it could be postulated that in the hypertrophying muscle, suppression of SSAT expression to elevate polyamine content would accumulate acetyl Co-A, thereby suppressing β-oxidation of fatty acid.

PAO transcription levels are known to be low in various cell types [33]. Although PAO induction by N1, N11-diethylnorspermine addition in human lung carcinoma cells has been reported [33], evidence of PAO induction in mammalian tissues has not yet been reported. Therefore, our finding that endurance exercise increases PAO protein levels in the plantar muscle provides new evidence for PAO induction.

### PAO localization in skeletal muscles

Confocal laser microscopy of the skeletal muscle fiber revealed that PAO was distributed in a striated pattern along the muscle fibers axis. To the best of our knowledge, the spatial expression of polyamine catabolic proteins within the skeletal muscle fiber has not yet been investigated, although spatial transcriptomic analysis of RNA expression of SSAT and SMOX at the whole-muscle level has been reported [14]. The localization of PAO was similar to that reported in the mitochondria [29], which structurally suggested close linkage between polyamine catabolism and aerobic metabolism.

PAO has been reported to localize in peroxisomes [34] and possess complex connections with mitochondria, such as degrading long-chain fatty acids that cannot be degraded by mitochondria and supplying acyl-CoA to mitochondria [35]. Our morphological analysis may further imply a close correlation between polyamine catabolism and mitochondrial function intertwined with peroxisomes.

### Effects of polyamine precursor administration in skeletal muscles

Putrescine administration did not induce appreciable changes in muscle volume or protein expression in skeletal muscles suggesting that putrescine is not an effective inducer of hypertrophy compared with exercise. However, the putrescine intake showed positive correlation with the protein expression of SPDSY (Fig.S1) and decreased the average protein expression of PGC−1α in the plantar muscles (Fig. 4A). As a hypothesis for the decrease in the PGC−1α related to putrescine intake in the plantar muscle, we consider the possibility that a decrease in SAM, a precursor of dcSAM, due to the increase in SPDSY suppresses the histone H3 lysine 4 trimethylation (H3K4me3) levels, reported as an active promoter mark, in the PGC1α promoter [36].

### Polyamine metabolism in the cardiac muscle

In the cardiac muscle, polyamine metabolic enzymes were minimally affected by the exercise and spermidine content decreased correlating with exercise volume despite the significant hypertrophic response. Whereas hypusinated form of eIF5A increased in a manner opposite to the change of spermidine content.

An earlier study has reported an increase in polyamine content of in the cardiac muscle after 3 months of treadmill exercise [37] or 5 days of swimming [38] in the male rats. Although we cannot discuss the different responses of polyamine from their studies and cause of the increase in eIF5A in this study, it may be inferred that polyamine levels in the cardiac muscle would be precisely regulated depending on the occasion to avoid unfavorable radial hypertrophy of muscle fiber, which could suppress systolic function of the heart [39]. The finding that putrescine administration increased AZ1 levels in the cardiac muscle may also suggest strict regulation of polyamine content in the cardiac muscle by inhibiting polyamine transport [40], [41] or ODC function.

## Limitations

A causal relationship between polyamine metabolism and muscle volume regulation was not verified. Experiments which determine whether inhibiting SSAT would eliminate the upregulation of PAO and induce muscle hypertrophy in fast-type muscles with monitoring amount of acetyl-CoA, for example, will be needed to verify the anticipation in this study.

## Abbreviations

ODC, ornithine decarboxylase

AZ, ornithine decarboxylase antizyme

AZ1, ornithine decarboxylase antizyme 1

SPDSY, spermidine synthase

SPMSY, spermine synthase

SMOX, spermine oxidase

dcSAM, decarboxylated S-adenosyl methionine

SAM, S-adenosyl methionine

SAMDC, *S*-adenosylmethionine decarboxylase

SSAT, spermidine/spermine N1-acetyltransferase

PAO, polyamine oxidase

mTOR, mammalian target of rapamycin

AMPKα, AMP-activated protein kinase α

PGC1α, peroxisome proliferator-activated receptor γ coactivator 1α

eIF4E, eukaryotic translation initiation factor 4E

eIF5A, eukaryotic initiation factor 5A

GLUT4, glucose transporter type 4

HK-2, hexokinase 2

CS, citrate synthase

COXIV, cytochrome oxidase complex IV

HADH, hydroxyacyl-coenzyme A dehydrogenase, mitochondrial

DRP1, dynamin−1-like protein

OPA1, mitochondrial dynamin-like GTPase

MCU, mitochondrial calcium uniporter

Acetyl-CoA, acetyl coenzyme A

## CRediT authorship contribution statement

**Kazuhiro Hirano:** Investigation. **Hideki Yamauchi:** Writing – review & editing, Supervision, Investigation, Formal analysis, Data curation, Conceptualization. **Michiaki Ikeda:** Writing – review & editing, Investigation. **Shigeru Morimoto:** Writing – review & editing, Investigation. **Toshiko Yamazawa:** Writing – review & editing, Investigation, Formal analysis, Data curation. **Naoya Nakahara:** Writing – review & editing, Investigation, Formal analysis, Data curation. **Makiko Ohkido:** Writing – review & editing, Investigation, Formal analysis, Data curation, Conceptualization. **Maki Yamaguchi:** Writing – review & editing, Writing – original draft, Investigation, Funding acquisition, Data curation, Conceptualization.

## Funding sources

This study was supported by Japan Society for the Promotion of Science KAKENHI (Grant Numner 25K14413) awarded to M. Yamaguchi.

## Declaration of Competing Interest

The authors declare the following financial interests/personal relationships which may be considered as potential competing interests: Maki Yamaguchi reports article publishing charges, equipment, drugs, or supplies, travel, and writing assistance were provided by Japan Society for the Promotion of Science. If there are other authors, they declare that they have no known competing financial interests or personal relationships that could have appeared to influence the work reported in this paper.

## Appendix A Supplementary material

Supplementary material

Figure S1 Sperman’s rank correlation efficient between measured parameters in this study with soleus, plantar, and cardiac muscles. Statistically significant values (p < 0.05) are shown

Figure S2 Muscle weight, putrescine and polyamine contents, averaged abundance of proteins after the 8-week of the experiment for groups of sedentary without putrescine application (Se/−), sedentary with putrescine application (Se/Pu), exercised without putrescine application (Ex/−), exercised with putrescine application (Ex/Pu) in the cardiac muscle. Data are indicated as means ± standard errors of the mean (S.E.M.). *, **, and *** indicate p < 0.05, p < 0.01, and p < 0.001 by two-way analysis of variance (ANOVA) for the factor of putrescine administration (for the putrescine content and abundance of AZ1) or exercise application (for others), respectively. Since two-way ANOVA indicated interaction between exercise and putrescine intake for the muscle volume, one-way ANOVA with post-hoc analysis by Tukey–Kramer method was performed and significant difference between Se/− vs Ex/Pu, Se/− vs Ex/−, Se/Pu vs Ex/− with p < 0.05, p < 0.01, and p < 0.01, respectively

## Footnotes

Appendix A Supplementary data associated with this article can be found in the online version at doi:10.1016/j.jphyss.2026.100083.
