# Supplementary material for: Contrasting response of polyamine metabolic enzymes underlying fiber-type specific volume regulation in the skeletal muscle with endurance exercise
Source: J Physiol Sci. 2026 Jun 17;76(2):100083. doi: 10.1016/j.jphyss.2026.100083 (PMC13314752; doi:10.1016/j.jphyss.2026.100083)
Supplement: Supplementary file 1 — Supplementary material [file mmc1.docx]

**Supplementary Table 1. Summary of body weight, fat rate, food intake, water intake, putrescine intake, and total running distance**

|  |  |  |  |  |  |  |  |  |
| --- | --- | --- | --- | --- | --- | --- | --- | --- |
|  |  | Exercise (-) | | Exercise (+) | | Two-way ANOVA | | |
|  |  | Putrescine (-) | Putrescine (+) | Putrescine (-) | Putrescine (+) | Exercise | Putrescine | Interaction |
| Body weight (g) | Before | 118.9 ± 1.92 | 120.9 ± 2.37 | 120.7 ± 2.46 | 122.2 ± 2.09 | ns | ns | ns |
|  | After | 199.2 ± 3.41* | 207.1 ± 4.36** | 193.6 ± 3.87 | 184.4 ± 2.26 | <0.001 | ns | <0.05 |
| Fat rate (%) | Before | 2.68 ± 0.51 | 3.21 ± 0.47 | 2.82 ± 0.42 | 3.24 ± 0.45 | ns | ns | ns |
|  | After | 29.61 ± 1.82 | 29.87 ± 1.34 | 20.29 ± 1.61 | 17.22 ± 1.33 | <0.001 | ns | ns |
| Food intake (g/day) |  | 12.3 ± 0.46 | 12.3 ± 0.47 | 13.7 ± 0.49 | 12.8 ± 0.46 | 0.06 | ns | ns |
| Water intake (ml/day) |  | 16.6 ± 0.70 | 16.6 ± 1.00 | 16.0 ± 0.77 | 15.6 ± 0.55 | ns | ns | ns |
| Putrescine intake (g/day) |  |  | 0.166 ± 0.01 |  | 0.156 ± 0.01 |  |  |  |
| Total running distance (km) |  |  |  | 571.25 ± 66.2 | 506.04 ± 103.9 |  |  |  |
|  |  |  |  |  |  |  |  |  |

Summary of body weight, fat rate, food, water, putrescine, and running distance. The sample numbers were 10, 9, 9, and 9 for putrescine without exercise, putrescine without exercise, putrescine with exercise, putrescine + without exercise, and putrescine + exercise, respectively. * and ** indicate significant difference compared with the exercise (+) putrescine (+) group by Tukey–Kramer post-hoc test with p < 0.05 and p < 0.01, respectively, after the two-way analysis of variance (ANOVA).

| **Supplementary Table 2. List of chemicals used for the analysisWestern blotting** |  |  |
| --- | --- | --- |
| **Target** | **Antibody** |  |
| Ornithine decarboxylase | E-6 (Santa Cruz sc-398116) |  |
| Ornithine decarboxylase antizyme 1 | RayBiotech 102-27282 |  |
| Spermidine synthase | Polyclonal antibody (Proteintech 19858-1-AP) |  |
| Spermine synthase | Polyclonal antibody (Proteintech 15979-1-AP) |  |
| S-adenosylmethionine decarboxylase | E-6 (Santa Cruz sc-166970) |  |
| Spermidine/spermine N1-acetyltransferase 1 | Polyclonal antibody (Proteintech 10708-1-AP) |  |
| Polyamine oxidase | Polyclonal antibody (Proteintech 18972-1-AP) |  |
| Spermine oxidase | Polyclonal antibody (Proteintech 15052-1-AP) |  |
| Phospho-AMP-activated protein kinase α (Thr172) | Cell signaling 2535 |  |
| Peroxisome proliferator-activated receptor γ coactivator 1α | Santa Cruz sc-13067 |  |
| Phospho-eukaryotic initiation factor 4E (Ser209) | Cell signaling 9741 |  |
| Hypusine ( = hypusinated eIF5A) | Polyclonal antibody (Millipore ABS1064) |  |
| Glucose transporter 4 | Millipore 07-1404 |  |
| Hexokinase 2 | Millipore AB3279 |  |
| Hydroxyacyl-coenzyme A dehydrogenase, β subunit | Sigma HPA037539 |  |
| Citrate synthase | Cell signaling 14309 |  |
| Cytochrome c oxidase complex IV | Cell signaling 4850 |  |
| Dynamin-related protein 1 | Cell ignaling 8570 |  |
| Optic atrophy 1 | Cell signaling 80471 |  |
| Mitochondrial calcium uniporter | Cell signaling 14997 |  |
| Horse anti-mouse IgG, HRP-linked secondary antibody | Cell signaling 7076 |  |
| Goat anti-rabbit IgG, HRP-linked secondary antibody | Cell signaling 7074 |  |
|  |  |  |
| **Immunostaining** |  |  |
| **Target** | **Antibody** |  |
| Polyamine oxidase | Polyclonal antibody (Proteintech 18972-1-AP) |  |
| Myomesin 1 | DSHB (mMac myomesin B4) |  |
| Goat anti-rabbit IgG Alexa fluor 647-conjugated | Jackson ImmunoResearch (111-605-144) |  |
| Goat anti-mouse IgG1, Dylight 405-conjugated | Jackson ImmunoResearch (115-475-205) |  |
| Nucleus | Propidium Iodide (Nacalai 19174-31) |  |
