# Supplementary figures and images for: Contrasting response of polyamine metabolic enzymes underlying fiber-type specific volume regulation in the skeletal muscle with endurance exercise
Source: J Physiol Sci. 2026 Jun 17;76(2):100083. doi: 10.1016/j.jphyss.2026.100083 (PMC13314752; doi:10.1016/j.jphyss.2026.100083)

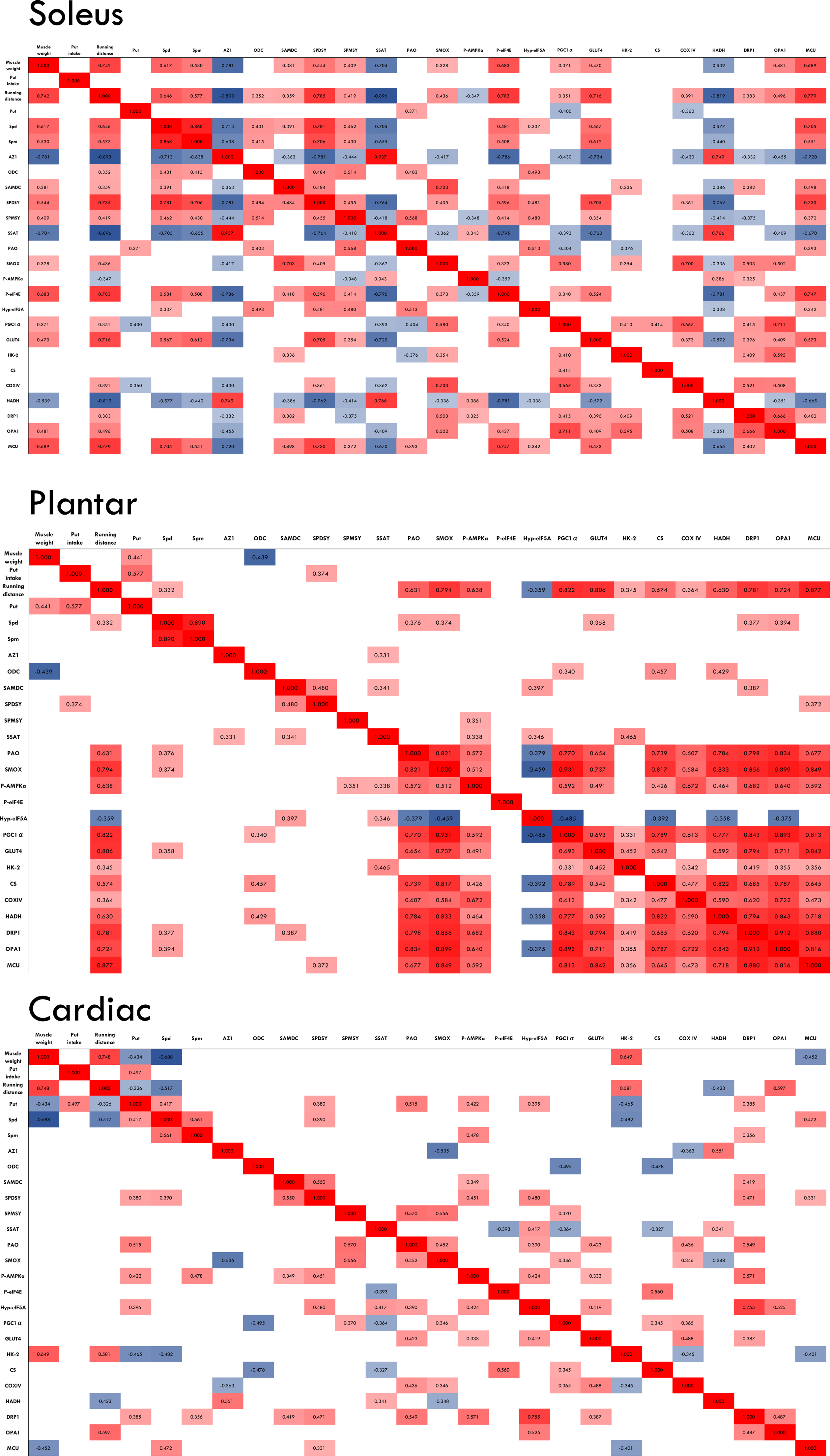

Supplement: Figure S1 — Sperman’s rank correlation efficient between measured parameters in this study with soleus, plantar, and cardiac muscles. Statistically significant values (p < 0.05) are shown [file mmc2.jpg]

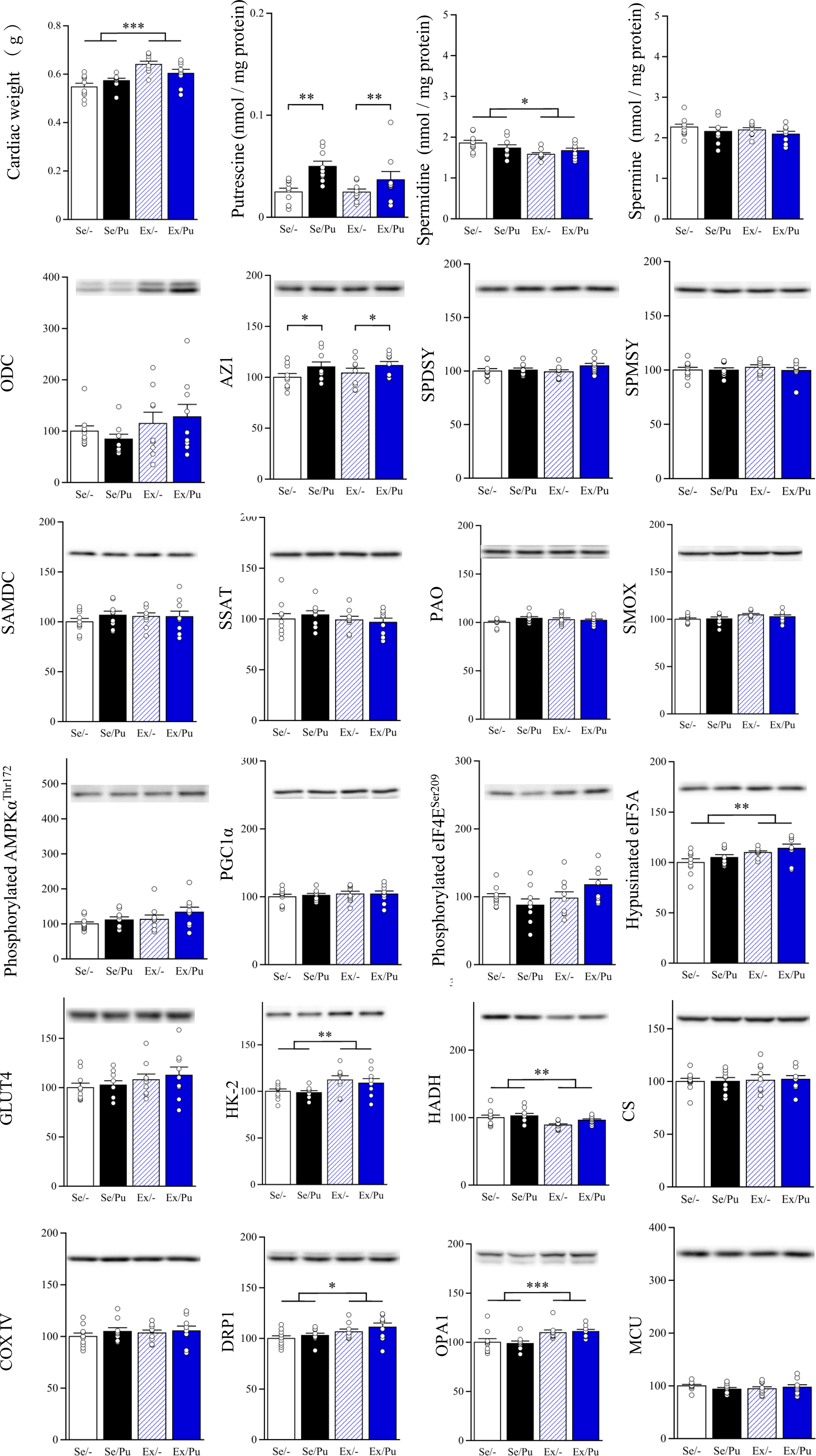

Supplement: Figure S2 — Muscle weight, putrescine and polyamine contents, averaged abundance of proteins after the 8-week of the experiment for groups of sedentary without putrescine application (Se/−), sedentary with putrescine application (Se/Pu), exercised without putrescine application (Ex/−), exercised with putrescine application (Ex/Pu) in the cardiac muscle. Data are indicated as means ± standard errors of the mean (S.E.M.). *, **, and *** indicate p < 0.05, p < 0.01, and p < 0.001 by two-way analysis of variance (ANOVA) for the factor of putrescine administration (for the putrescine content and abundance of AZ1) or exercise application (for others), respectively. Since two-way ANOVA indicated interaction between exercise and putrescine intake for the muscle volume, one-way ANOVA with post-hoc analysis by Tukey–Kramer method was performed and significant difference between Se/− vs Ex/Pu, Se/− vs Ex/−, Se/Pu vs Ex/− with p < 0.05, p < 0.01, and p < 0.01, respectively [file mmc3.jpg]
